# Subacute Peristomal Necrotizing Fasciitis Detected During Adjuvant Chemotherapy for Adenocarcinoma Rectum: Case Report on a Unique Presentation and Description of a Simple Surgical Strategy for Treatment

**DOI:** 10.7759/cureus.2075

**Published:** 2018-01-16

**Authors:** Humaid Ahmad, Jahanzaib Haider, Sheeraz S Siddiqui, Sumbul Naz, Faizan Nihal, Shams Nadeem Alam

**Affiliations:** 1 Department of Surgery / Hepatopancreatobiliary and Liver Transplant Unit, Dow University of Health Sciences (DUHS), Karachi, Pakistan; 2 Department of Surgery, Dow University of Health Sciences (DUHS), Karachi, Pakistan; 3 Hepatopancreatobiliary and Liver Transplant Unit, Dow University of Health Sciences (DUHS), Karachi, Pakistan

**Keywords:** peristomal necrotizing fasciitis, necrotizing fasciitis, subacute necrotizing fasciitis, peristomal complications, chemotherapy complications

## Abstract

Necrotizing fasciitis (NF) is a rare entity with its subacute form being even rarer. The condition has also been described to occur in the peristomal area in relation to different clinical scenarios. However, it has not been described in the setting of adjuvant chemotherapy where these medications have already been given. While the diagnosis may be delayed or missed due to the subtle symptomatology of the indolent subacute form of NF, another reason for a delayed or missed diagnosis may be the excessive use of tape and the stoma belt around the stomal appliance to prolong the life of the appliance beyond its recommended days of usage due to economic constraints. This, in turn, covers a larger area around the peristomal skin and developing skin changes associated with necrotizing fasciitis. Despite the less aggressive presentation of the subacute form, it may still represent a unique surgical challenge in this setting, as the chemotherapy-induced neutropenia bound to occur a few days after the chemotherapy may expose the patient to serious complications at that time. As such, the surgical plan at the time of presentation may become the determinant of morbidity and mortality. Here, a case is presented of a young patient who underwent abdominoperineal resection for stage III adenocarcinoma of the rectum. He was referred to us on the day of the fourth cycle of adjuvant chemotherapy by the oncology service where he had received part of his chemotherapy regimen. On presentation, he was found to be having significant skin changes in the peristomal area consistent with necrotizing fasciitis despite being clinically stable. The authors present this unique case as a study from which many lessons can be learned. They also explain the thought process behind a well-planned but simple surgical strategy that was implemented with a successful patient outcome. In addition to describing this surgical strategy, the case report is concluded by highlighting some factors that may raise suspicion of this condition and by emphasizing routine history-taking for peristomal symptomatology and a thorough examination of the peristomal site prior to administration of chemotherapy in patients with stomata, as this condition, if overlooked, may lead to a fatal outcome.

## Introduction

Necrotizing fasciitis (NF) is a rare, but potentially fatal infection involving the skin, subcutaneous tissue, and fascia [1]. It is also known as flesh-eating disease and causes death that can be sudden and dramatic [1]. It is a condition that tends to occur in patients with an immunocompromised state [2]. Although the clinical presentation of NF includes a combination of severe local symptoms and systemic disturbances, concern has been generated regarding a subacute indolent form of the disease, where the patient presents initially with minimal symptomatology [1]. This subtle presentation can lead to the diagnosis being delayed or missed with a worse clinical outcome, as the disease can spread undetected until larger areas of skin and subcutaneous tissue are involved, resulting in greater tissue loss [1]. While patients with stomata can develop many peristomal skin complications, ranging from dermatitis to peristomal ulceration, NF in the area adjacent to the stoma (i.e. peristomal NF) has rarely been reported to occur [3-8]. Different clinical scenarios with peristomal NF have been reported [3-8], however, an extensive literature search did not reveal this entity being described in the setting of adjuvant chemotherapy, especially in a case where these medications had already been given. This setting in itself presents a unique clinical problem because chemotherapy-induced neutropenia a few days after dosage can exponentially increase the risk of serious complications in a patient with a disease such as NF. The authors present a case of this situation where extensive peristomal NF was detected during dosage of the patient's adjuvant chemotherapy regimen. The case illustrates the difficulty in diagnosis that clinicians can face when dealing with a subacute form of NF and is also presented to describe the carefully planned, but simple, surgical strategy that was employed in view of the anticipated chemotherapy-induced neutropenia and its related potential for serious complications. As such, the case represents important lessons to be learned and especially bears importance for surgeons as well as oncologists.

## Case presentation

A 24-year-old, otherwise healthy, male patient with chronic hepatitis B and a Child-Pugh Score of 5 (Class A) underwent abdominoperineal resection for stage III adenocarcinoma of the rectum at our department. He had a prolonged postoperative course due to perineal wound infection, but was finally discharged after 76 days of admission and referred for adjuvant chemotherapy.

**Figure 1:**
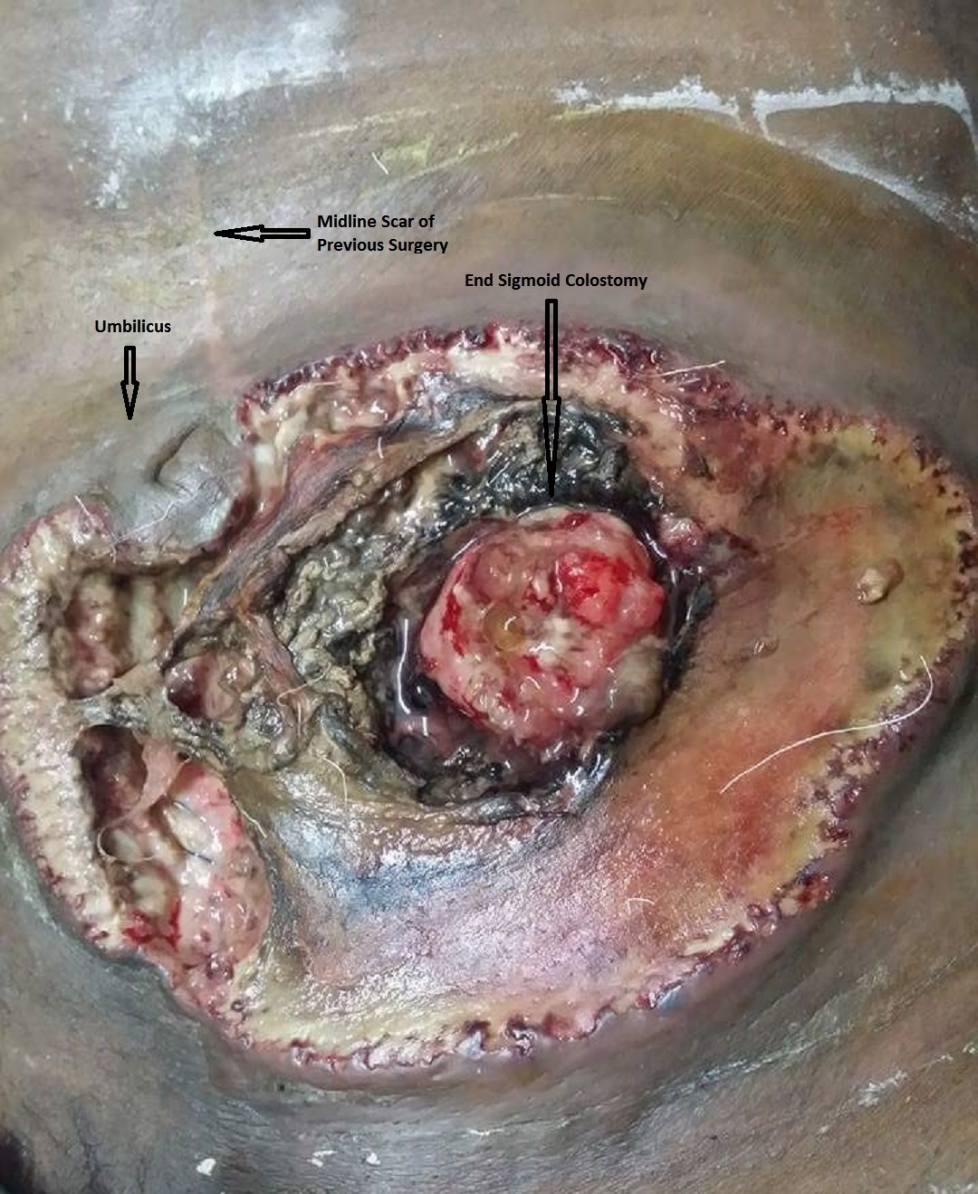
Peristomal necrotizing fasciitis - findings on initial presentation Extensive necrosis of the skin and subcutaneous tissue around the end-sigmoid colostomy site that extends across the midline medially. The uninvolved umbilicus is visible at the upper-right edge of this circumferential area with the scar of a previously healed incision above the umbilicus.

The patient presented on the day of his fourth cycle of chemotherapy. He was on the FOLFOX-4 regimen. He had received part of his cycle earlier in the morning, which included intravenous (IV) boluses of 5-fluorouracil, oxaliplatin, and leucovorin. During this treatment, he complained of having mild discomfort and irritation in the area of his stoma. Upon removal of his stoma appliance, the oncology service noted an area of necrotic skin around his stoma. He was urgently referred back to us for management. Upon arrival, the patient was alert and oriented. He was vitally stable and not in sepsis. He complained of only mild discomfort around his stoma site for the past few days. He had been using the same stoma appliance for well over a month. As he had only minimal symptoms, he did not mention these symptoms at initial presentation to the oncology service. His systemic examination was unremarkable. On examination of his abdomen, a wide area of necrotic skin and subcutaneous fat was seen extending around his end-sigmoid colostomy up to his umbilicus superomedially. The colon at the stoma site was thickened and inflamed (Figure 1). Close clinical examination revealed that the colon at the stoma site adhered very well to his abdominal wall with no dehiscence despite the overlying findings and that the necrosis had not spread beyond the deep fascia to involve the abdominal wall musculature. The rest of his abdominal examination was unremarkable, with a well-healed midline laparotomy scar of his previous surgery. The patient’s baseline labs were normal, including his total leucocyte count.

As debridement was necessary and chemotherapy-induced neutropenia was anticipated after a few days, a carefully planned surgical approach was used to lessen the chances of complications during the neutropenia phase. The patient was given general anesthesia and a loop transverse colostomy was first constructed in the right hypochondrium after pulling the transverse colon through a small incision. Following this, debridement of the area of necrosis was undertaken down to the musculature, leaving an erythematous outer edge. The end-sigmoid colostomy was not taken down and its opening was closed. The two steps of using a small incision in the right hypochondrium to create the transverse colostomy rather than a midline incision and closing the end-sigmoid colostomy opening without taking the stoma down were done to avoid extra incisions and prevent even the least contamination of the peritoneal cavity. Such contamination could have occurred from the infected area (area of peristomal NF) or from colonic contents that could be spilled into the midline wound or peritoneal cavity during the dissection and mobilization of the colon. The thought process behind this approach was as follows:

While deep surgical site infection and wound infection related to such contamination may not have severe consequences at the time of surgery, as the patient was immunocompetent, the chances of flare up and resultant sepsis a few days later during the chemotherapy-induced neutropenia phase could lead to severe complications and a fatal outcome at that point in time.

**Figure 2:**
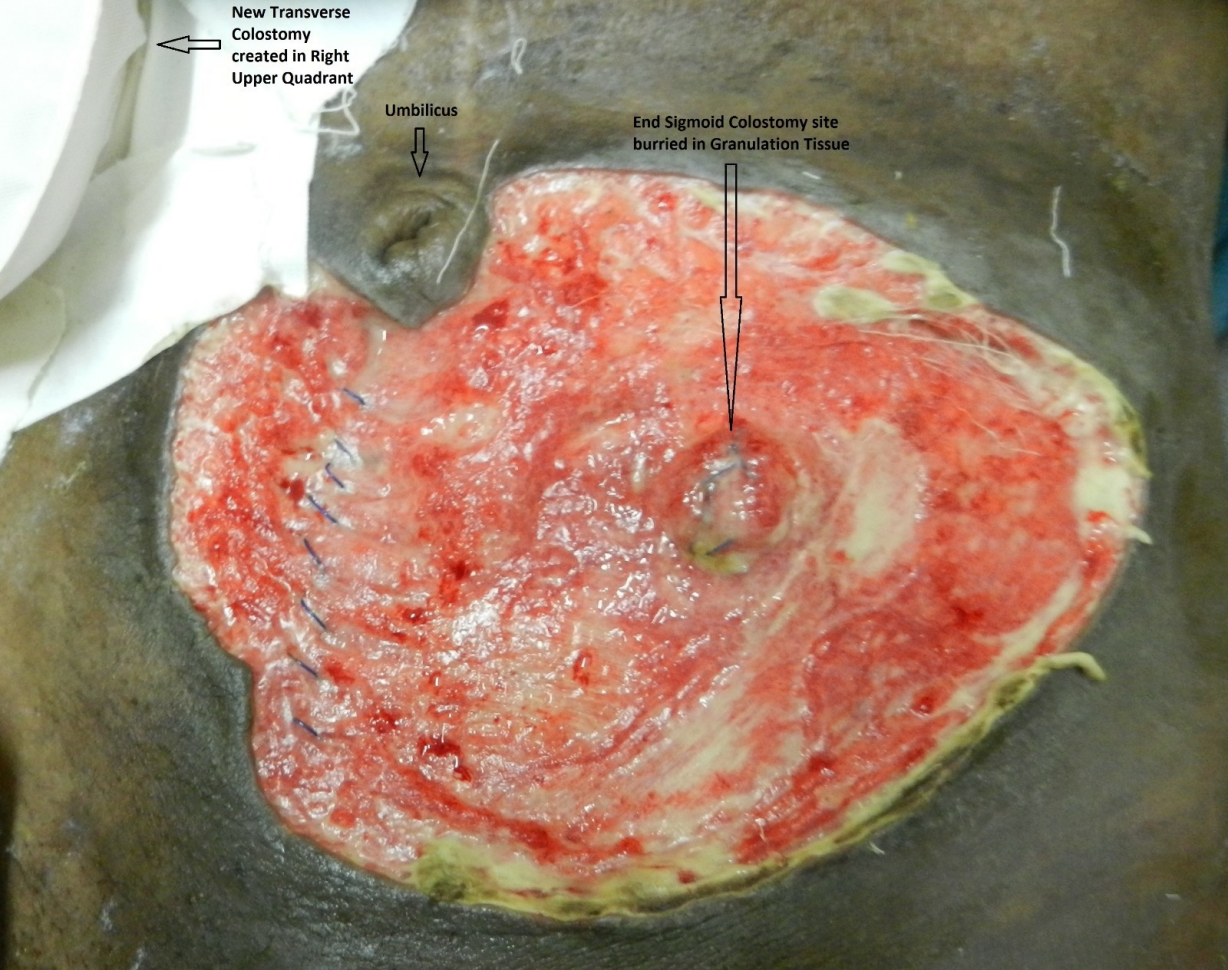
Fourth postoperative day of debridement The outcome on the fourth postoperative day of the initial debridement. The end- sigmoid colostomy site is buried in the dirty granulation tissue, which has now filled the entire defect.

As the end-sigmoid colostomy site was also thickened due to the inflammatory process, three punch biopsies were taken to rule out recurrence. These biopsies were subsequently negative for malignancy. Postoperatively, the patient was kept in the ward where he was given IV broad-spectrum antibiotics and IV anti-fungal agents with daily dressings of the debrided site. Subcutaneous filgrastim was started on the third postoperative day (pod). On the fourth pod, the previously closed end-sigmoid colostomy dehisced (Figure 2). Re-suturing was attempted but was unsuccessful due to friable tissue. As the newly constructed proximal transverse loop colostomy had started working from the second pod and the end-sigmoid colostomy discharged only mucus, it was decided to continue with daily dressings of this area.

As anticipated, the patient developed grade 4 neutropenia (counts less than 500 cells/mm^3^) by the seventh pod. The patient had a single episode of high-grade fever during this time, however, he did not develop any other signs of sepsis. He was continued on the same regimen with strict observation and a repeat of daily routine laboratory tests. His counts gradually improved and became normal by the 12^th^ pod.

The patient underwent an exploratory laparotomy three weeks later for resection of his distal colon, including his end-sigmoid colostomy site, with the plan of making the transverse colostomy his permanent stoma. Although the initial punch biopsies were negative for malignancy, this option was selected, as a computed tomography (CT) scan performed during admission showed a thickening of the end-sigmoid colostomy with a suspicion of recurrence below the abdominal wall. Endoscopy was not planned preoperatively due to the risk of perforation at the friable end-sigmoid colostomy site. The patient also had a FibroScan (Echosens, Paris, France) prior to the procedure due to a history of chronic hepatitis B. This showed a Metavir score of 4.9 that was consistent with the F0-F1 stage of fibrosis.

**Figure 3:**
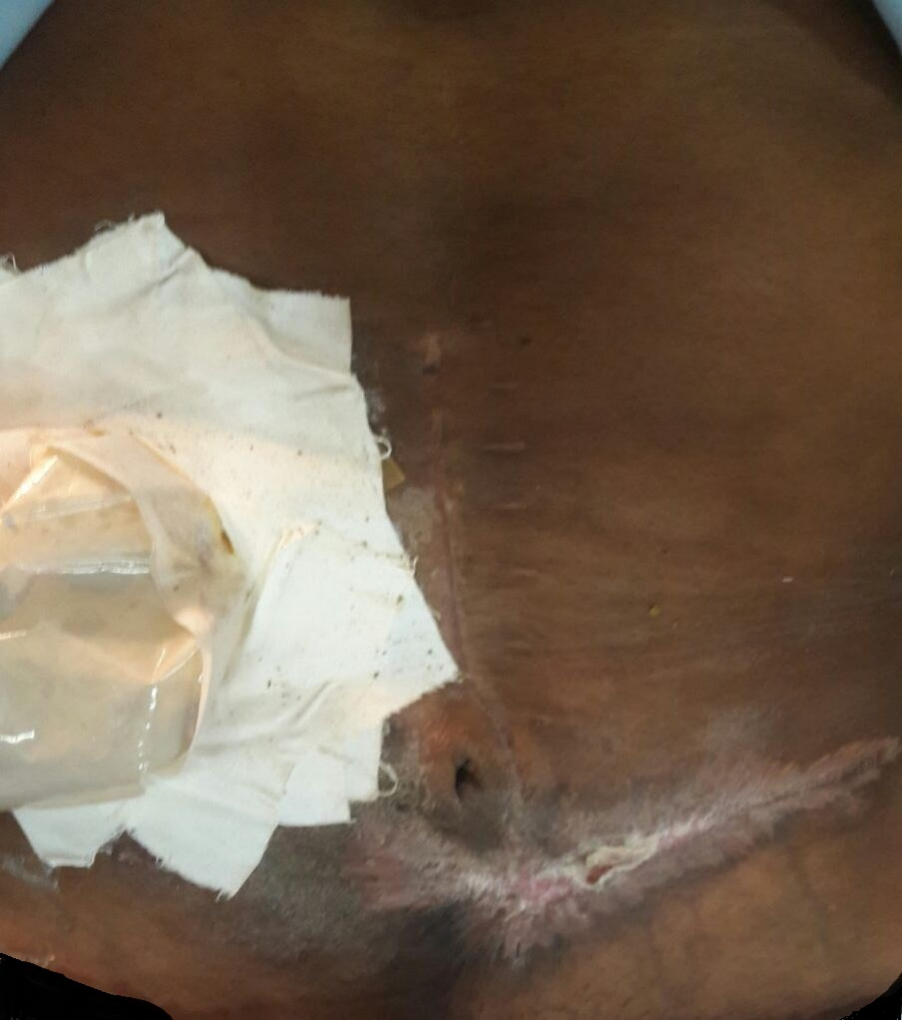
Four months after initial debridement The outcome four months after the initial debridement. There is a healed scar at the debridement site in the left-lower quadrant of the abdomen.

Postoperatively, the patient remained well. He was discharged on the 10^th^ pod of his second surgery and urgently referred for completion of his adjuvant chemotherapy. Histopathology of the resected specimen did not reveal any recurrence of his cancer. The patient followed up four months later with his wound completely healed (Figure 3).

## Discussion

This case presents a very rare and unique situation. Multiple factors interplayed to produce a complex problem that started with the initiation and development of the disease process, the involvement of a wide area of soft tissue, a delay or miss in diagnosis, the start of treatment, and finally an end with a good patient outcome. The most relevant of all these issues is why the diagnosis was delayed or missed and what should be the important considerations when planning treatment in such a situation. The authors try to elaborate on all these issues.

NF is a rare condition [5] that usually holds a fatal outcome and occurs in an immunocompromised state [1]. Some of the factors that cause an immunocompromised state and are relevant to our patient include liver cirrhosis [2] and drugs that cause immunosuppression by virtue of creating neutropenic states [2,8]. As our patient had a Metavir score of 4.9 consistent with the F0-F1 stage of fibrosis on FibroScan, despite having chronic hepatitis B, liver cirrhosis was virtually ruled out. We, therefore, felt that chemotherapy-induced neutropenia was the main factor behind the initiation of the disease process.

While peristomal NF has been rarely reported in small series or case reports [3,8], stomata can lead to NF due to peristomal inflammation [5]. Possible triggering factors in peristomal NF are skin abrasions from stoma appliances or excoriation due to stomal secretions [8]. These skin injuries, in turn, create a pathway for bacterial entry [8] that leads to the irritation and inflammation of the peristomal area. Protection of the peristomal skin can avoid such complications and is undertaken by applying a tightly fitting stoma appliance and changing it regularly once it becomes inefficient.

However, it is unfortunate that economic constraints in our part of the world force patients to use stoma appliances well beyond their proposed time of usage. It is quite a common observation in our practice that this is done by anchoring them using belts made out of easily available cheap rubber or by applying more and more zinc oxide tape around the appliance; techniques which were also being practiced by our patient for the same reasons. Such techniques additionally cover a larger area of peristomal skin where infection and inflammation may be ongoing. While these techniques seem economically feasible, they are used at the expense of an inadequately created seal that is required to protect the peristomal skin.

As the diagnosis of peristomal NF in our patient was made during the first day of dosage of the fourth cycle of chemotherapy, a point in time when patients have recovered from the neutropenia phase, we feel that the process of peristomal NF could have started during the neutropenia phase of the previous chemotherapy cycle. This process, in turn, spread during the rest period of the third chemotherapy cycle. The use of an inefficient stoma appliance for well over a month seems to have allowed an environment where skin damage and bacterial entry became possible with neutropenia-induced immunosuppression causing severe infection and inflammation. As the peristomal skin was heavily covered by excessive tape, a large area of skin got involved before being identified. This seems to be the only plausible explanation, as no other etiological factors for NF were identifiable in our patient.

The above discussion still does not explain how such widespread NF occurred without the patient noticing any clinical changes. The reason seems to be the subacute form of NF. While NF is a condition that is recognized as a cause of severe clinical toxicity and systemic upset, a rather subtle presentation has also been described [1,9]. In this form, the patient presents with minimal symptomatology until a sudden clinical deterioration occurs [1,9]. This subacute form of NF presents an even rarer and less clearly defined clinical entity [1,9]. Wong et al. [9], after a careful Medline search, proposed a diagnostic criterion for this subacute form in order to differentiate it from other more aggressive forms of NF. The clinical part of this criterion includes an indolent initial course with the absence of systemic disturbances, the gradual progression of tissue necrosis with progressive cutaneous changes over the affected site, progression despite the use of antimicrobials, and a sudden deterioration with rapid progression of NF or the systemic features of sepsis. The first two clinical criteria mentioned by Wong et al. are similar to the presentation in our patient, thus making this an even rarer clinical scenario.

As such, through this case, we identify multiple factors that led to a widespread disease and a delayed or missed diagnosis. These include the rather subtle symptomatology of presentation, economic constraints, the overuse of an inefficient stoma appliance, and possibly the rest period of the chemotherapy cycle when patients are relatively unattended.

As far as treatment is concerned, early surgical debridement is considered the cornerstone of treatment for NF [1,9]. While the indolent course of subacute NF may be a favorable situation in the early period, at what stage will rapid progression occur remains less clear [9]. In the review by Wong et al. [9], a wide range from six days to more than a month was found. Therefore, early debridement is recommended regardless of presentation and of whether it is the subtle subacute form or the more accelerated forms of NF [9].

In peristomal NF, an important clinical question that arises is whether the stoma site should be repositioned or not. Knowledge derived from the study of perianal wounds has shown that the passage of stools does not modify the evolution of the wound healing process [10]. This knowledge has even been extrapolated to avoid stomal repositioning in severe peristomal infections by at least one treating physician [10]. However, our patient presented an additional clinical problem, as chemotherapy-induced neutropenia was bound to occur in our patient a few days later. During this neutropenia phase, how far the immune system would be able to counter microorganisms in feces was questionable for us. This compelled us to decide that stomal repositioning should be done, as fecal contamination of the debrided area at that point in time could become the source of severe sepsis, morbidity, and mortality. Conversely, another point we had to consider was that stomal repositioning would mean requiring major surgery with a midline laparotomy incision and exposure of the incision as well as the peritoneal cavity to possible fecal contamination during colon mobilization. Even if fecal contamination was prevented by a meticulous technique, the exposure of the peritoneal cavity or midline incision to the peristomal necrotic tissue could still lead to severe sepsis later on during the neutropenia phase. Therefore, a simple surgical approach was applied, which included the construction of a proximal stoma through a small incision well away from the peristomal NF site followed by debridement of the NF area and closure of the original stoma site. This decision was specifically based on our initial careful clinical examination, which revealed the bowel-abdominal wall musculature interface at the stoma site to be well healed and intact. The end result of this surgical strategy was avoidance of a major procedure and unnecessary incisions, prevention of peritoneal contamination, and a much less anesthesia time.

## Conclusions

While this case presents a very rare and unique scenario, it represents a situation that compels clinicians to cultivate certain guidelines. In cancer patients with stomata, a careful history of peristomal symptomatology and an examination of the stoma site should be carried out at the time of presentation for chemotherapy. This is emphasized because although this condition is very rare, it can lead to serious consequences and may be missed, particularly because of the subacute presentation. The economic constraints of patients, the overuse of a stoma appliance beyond the recommended days of usage, and evidence of the use of methods to excessively anchor the stoma appliance should especially prompt this approach. In the event that peristomal NF is diagnosed in such a clinical situation as this patient, the simple surgical strategy described may be the most feasible treatment option. The urgent creation of a proximal stoma away from the infected area followed by surgical debridement without stoma takedown can obviate the need for major surgery and extra incisions, which may become the source of infection, morbidity, and mortality during the chemotherapy-induced neutropenia phase. This decision should, in turn, be guided by a thorough clinical examination of the infected area, which should reveal that the disease process has not affected the bowel-abdominal wall musculature interface at the stoma site. As such, this simple surgical strategy may prove life-saving.
